# Enhancing Intrinsic Stability of Hybrid Perovskite Solar Cell by Strong, yet Balanced, Electronic Coupling

**DOI:** 10.1038/srep30305

**Published:** 2016-07-26

**Authors:** Fedwa El-Mellouhi, El Tayeb Bentria, Sergey N. Rashkeev, Sabre Kais, Fahhad H. Alharbi

**Affiliations:** 1 Qatar Environment and Energy research Institute (QEERI), Hamad Bin Khalifa University, Doha, Qatar; 2 College of Science and Engineering, Hamad Bin Khalifa University, Doha, Qatar; 3 Department of Chemistry, Physics, and Birck Nanotechnology Center, Purdue University, West Lafayette, Indiana 47907, USA

## Abstract

In the past few years, the meteoric development of hybrid organic–inorganic perovskite solar cells (PSC) astonished the community. The efficiency has already reached the level needed for commercialization; however, the instability hinders its deployment on the market. Here, we report a mechanism to chemically stabilize PSC absorbers. We propose to replace the widely used methylammonium cation (CH_3_NH_3_^+^) by alternative molecular cations allowing an enhanced electronic coupling between the cation and the PbI_6_ octahedra while maintaining the band gap energy within the suitable range for solar cells. The mechanism exploits establishing a balance between the electronegativity of the materials’ constituents and the resulting ionic electrostatic interactions. The calculations demonstrate the concept of enhancing the electronic coupling, and hence the stability, by exploring the stabilizing features of CH_3_PH_3_^+^, CH_3_SH_2_^+^, and SH_3_^+^ cations, among several other possible candidates. Chemical stability enhancement hence results from a strong, yet balanced, electronic coupling between the cation and the halides in the octahedron. This shall unlock the hindering instability problem for PSCs and allow them to hit the market as a serious low-cost competitor to silicon based solar cell technologies.

In the past few years, the solar cell community has witnessed an exceptional emergence of a new family of solar cell materials^1^,^2^,^3^; namely hybrid perovskite solar cells (PSC). Within just four years, the conversion efficiency has ramped up dramatically and it is now above 20%^4^. This dramatic development is believed to be a result of a unique supportive combination of different properties of these materials, including the favorable balance between strong absorption and long carrier lifetime^5^, the efficient transport^6^,^7^,^8^, and the benign fault tolerance^9^. From a practical perspective, it is also impressive how simple to fabricate the PSCs and how many efficient cells made with various hybrid perovskites absorbers (the mostly used compound of this family is CH_3_NH_3_PbI_3_) and with different device designs. The only remaining obstacle before large scale commercialization is the cells instability^1^,^2^,^3^.

Currently, it is well known that CH_3_NH_3_PbI_3_ is not stable; this is due to many extrinsic and intrinsic causes. Extrinsically, it is sensitive to moisture, UV exposure, and oxygen^3^,^10^. Another important aspect that contributes significantly to the instability is the moderate crystal quality^11^, that ignites consequently an additional mixture of instability issues. Moreover, there is a debate about the severity of the dynamics of the polarized molecular cations such as CH_3_NH_3_^+^^12^,^13^, believed now to contribute to many materials related characters. More fundamentally, CH_3_NH_3_PbI_3_ suffers from intrinsic instability that can result in disorder and hence larger defect density, assist phase transition, and make the materials thermally active. Recently, Zhang *et al*.^10^ confirm computationally that the compound is thermodynamically unstable and suggested the existence of a kinetic barrier that prevents its spontaneous decomposition to CH_3_NH_3_I and PbI_2_; however, the decomposition is inevitable in the long-term.

Structure-wise, the 3-dimensional (3D) CH_3_NH_3_PbI_3_ is composed of a network of corner-sharing PbI_6_ octahedra and molecular cations (CH_3_NH_3_^+^) hosted between the cages. The main features of the resulting electronic structure are: 1) the top of valance band is composed of the 5*p* orbitals of the iodine, 2) the edge of the conduction band is formed from the 6*p* orbitals of the lead, and 3) the electronic states due to CH_3_NH_3_^+^ are located several electonvolts above and below the band gap edges and they don’t contribute directly neither to the optical properties within the solar spectrum range nor to the electronic transport^5^,^14^. These facts entail many other consequences: first, the cation can be used “indirectly” to tune the optical and electrical properties by distorting the octahedral network. Filip *et al*.^14^ and Knutson *et al*.^15^ utilized this concept to tune the gap in 3D and 2D hybrid perovskites respectively. Secondly, the cohesion within the crystal between PbI_6_ octahedra and CH_3_NH_3_^+^ is mainly due to weak electrostatic interactions^16^ as the electronic coupling between the octahedron and the cation is negligible. Thus, it was found that the cohesion is relatively weak^3^,^16^ as characterized by the relatively small site Madelung potential^16^,^17^ leading to chemical instability.

Currently, molecular cation design and substitution approach is an active area of research where the focus has been almost utterly on tuning the optoelectronic properties^14^,^17^,^18^. While, this could help in further enhancing the efficiency of PSCs; their commercial deployment is on hold till instability issues are resolved. Here, we demonstrate that the cation design can be used as a mechanism to enhance the stability by triggering stronger electronic coupling and electrostatic interactions like hydrogen-bonding, halogen-bonding, and van der Waals. This constitutes a revival of mechanisms routinely utilized other disciplines such as for the construction of polymers and metal Organic frameworks^19^,^20^,^21^,^22^ to the world of PSC.

In this work, we investigated computationally using density functional theory (DFT), the possibility of manipulating the electronic coupling between the molecular cation and PbI_6_ octohedron to enhance the stability of hybrid perovskites materials. This question has been investigated experimentally mainly by mixing CH_3_NH_3_^+^ with CH(NH_2_)_2_^+^ and Cs^+^^23^,^24^. Detailed structural investigations show that the cations’ mixing is not homogeneous; but rather a mix of grains of different materials^25^. Actually, this can be concluded as well from the XRD data in ref. 24. However, it turns out that this mix of grains limits ion migration^25^ which is one of the instability causes. Nevertheless, higher cation mixing was achieved^26^,^27^; but not used to improve solar cell stability. A recent report^28^ investigated the structural stability of CH(NH_2_)_2_^+^ and Cs^+^ and their mixtures using a revised geometrical tolerance factor in a great detail indicates that the mixing should allow better structural stability. The commonly used CH_3_NH_3_^+^ cation is composed of the strong electronegative atom of N and the moderate electronegative atom of C. By considering the electronegativity of I, the hydrogen atoms are generally tightly bonded to CH_3_NH_3_^+^ making its interaction with the PbI_6_ octahedron fairly small. By replacing the N atom by a less electronegative atom, the binding of the hydrogen atoms to the cation can be reduced offering the possibility to enhance the electronic coupling with PbI_6_ octahedron. We found that this indeed improves the chemical stability considerably, which is quantified by the reaction^29^ and the hull^30^,^31^,^32^ energies. The reaction energy is the difference between the total energy of a reaction and reactants, whereas the hull energy is the difference in formation energies and it effectively evaluates the stability of a given compound against any linear combination. While many possibilities for phase separation exist; we used the separation into the most stable binary and ternary compounds based on phase diagrams constructed from the Materials’ Project database^33^.

For sake of systematic analysis, we focused only of the tetragonal phases at which CH_3_NH_3_PbI_3_ crystallizes at room temperature. Also, we consider the two other commonly used cations for PSC; i.e., Cs^+^ and CH(NH_2_)_2_^+^. Our DFT stability calculations show that all CH_3_NH_3_PbI_3_, CH_3_NH_3_PbBr_3_, CH(NH_2_)2PbI_3_, and CsPbI_3_ are marginally stable in agreement with previous studies^10^,^34^. By replacing the molecular cation with the purpose of enhancing the electronic coupling between the cations and PbI_6_ octahedra, we found that CH_3_PH_3_^+^, CH_3_SH_2_^+^, and SH_3_^+^ cations result in more stable hybrid perovskite crystals while maintaining the suitable energy gap for solar cells. The only exception is CH_3_PH_3_PbBr_3_, where the interaction (P-H-Br) becomes extremely strong causing the bridging bond (P-H) elongation by 10% and unbalancing of the interactions and chemical destabilization of material. The strength of the electronic coupling is accessed from electronic structure and electronic density analysis, measures of relevant bond elongation, and electronic delocalization given in terms of computed normalized participation ratio (PR).

## Results & Discussion

**Figure 1:**
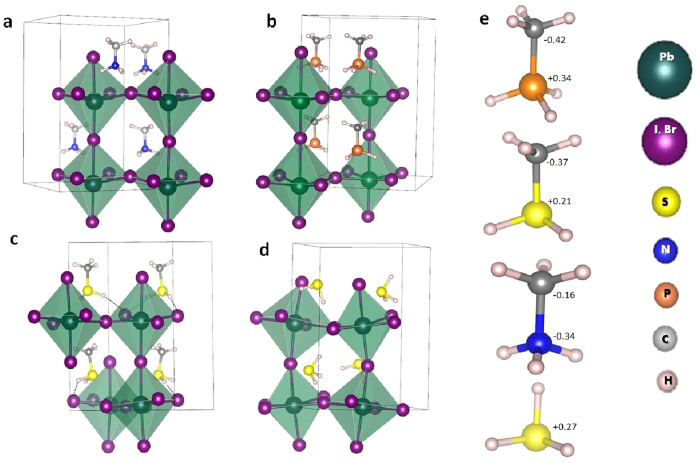
Crystal structures of the tetragonal phases of (**a**) CH_3_NH_3_PbX_3_, (**b**) CH_3_PH_3_PbX_3_, (**c**) CH_3_SH_2_PbX_3_, and (**d**) SH_3_PbX_3_ where X = I or Br. (**e**) The partial charges of the used cations^36^,^37^.

**Figure 2:**
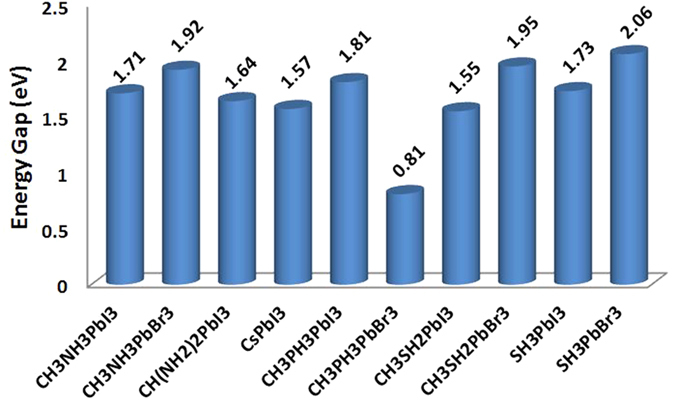
The calculated band gaps of the tetragonal phases of the considered materials in this work.

As we aim to replace the methylammonium cation in CH_3_NH_3_PbI_3_ and CH_3_NH_3_PbBr_3_ to improve the stability without deteriorating the suitable energy gap (*E*_*g*_), the cation must be relatively small to maintain the 3D dimensionality of the hybrid perovskites and must not contain any of the high electronegative elements to allow dragging (binding) one of its H atoms more toward the PbI_6_ or PbBr_6_ octahedron. There are many possible cations satisfying this condition and not limited to the ones considered in this work, namely CH_3_PH_3_^+^, CH_3_SH_2_^+^, and SH_3_^+^. The relaxed crystal structures in tetragonal phases with the new cations are shown in Fig. 1. The calculated gaps of these materials beside those of tetragonal phases of CH(NH_2_)2PbI_3_, and CsPbI_3_ are shown in Fig. 2. Clearly, the band gap is slightly affected by cation substitution, but remains suitable for solar cell applications except for CH_3_PH_3_PbBr_3_, where it is reduced considerably due to the emergence of an intermediate state resulting from a formed bond between Br, the bridging H atom, and P^35^. As will be shown shortly, this results in losing the electrostatic balance and hence deteriorating the electronic coupling and chemical stability.

**Figure 3:**
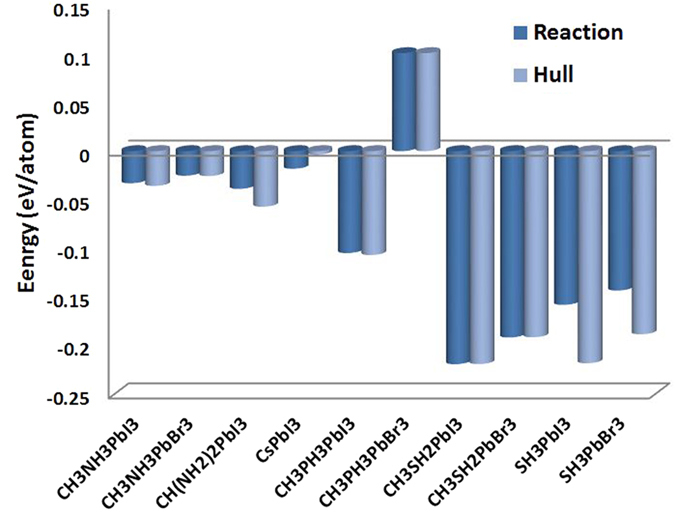
The calculated reaction and hull energies of the phase separation reactions of the considered materials to the most stable compounds.

As explained in the Methods section, the phase diagrams are generated for a given stoichiometry. Then, we deduce from the generated phase diagrams the decomposition route to the most stable binary and ternary compounds. Hereunder listed are the phase separation reactions of the considered materials to the most stable compounds. The calculated reaction and hull energies of these reactions are shown in Fig. 3.

1. CH_3_NH_3_PbI_3_ → NH_4_I + H_2_C + PbI_2_
2. CH_3_NH_3_PbBr_3_ → NH_4_Br + H_2_C + PbBr_2_
3. CsPbI_3_ → CsI + PbI_2_
4. 3CH(NH_2_)_2_PbI_3_ → 3 NH_4_I + NH_3_ + 3PbI_2_ + N_2_ + 3C
5. CH_3_PH_3_PbI_3_ → PH_4_I + H_2_C + PbI_2_
6. CH_3_PH_3_PbBr_3_ → PH_4_Br + H_2_C + PbBr_2_
7. CH_3_SH_2_PbI_3_ → PbS + H_2_C + 3HI
8. CH_3_SH_2_PbBr_3_ → PbS + H_2_C + 3HBr
9. SH_3_PbI_3_ → PbS + 3HI
10. SH_3_PbBr_3_ → PbS + 3HBr

The calculations show that CH_3_PH_3_PbBr_3_ is not stable (positive energies). As will be shown shortly, this is due to the lose of electrostatic balance. Also, CH_3_NH_3_PbI_3_, CH_3_NH_3_PbBr_3_, CH(NH_2_)2PbI_3_, and CsPbI_3_ are marginally stable as the calculated reaction and hull energies are between −100 and 0 meV/atom, which are the assumed sensitivity margin (please check the Methods section). Here the considered phase for CsPbI_3_ is alpha phase. But, it is known – and as confirmed the calculations (Supplementary materials)– that the delta phase is more stable. But, its energy gap is 2.54 eV which is unsuitable of solar cells.

**Figure 4:**
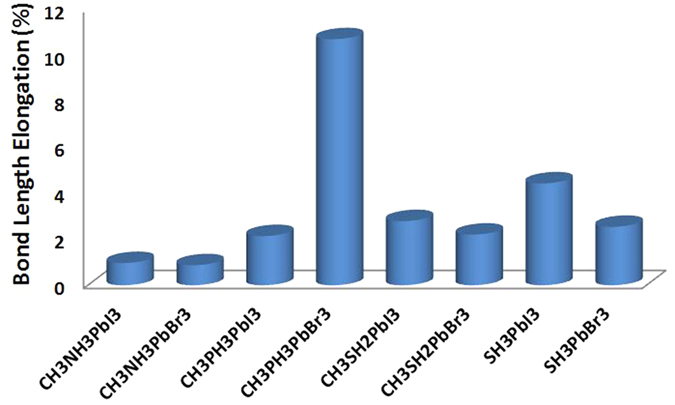
The calculated bond length elongation between the bridging H and its atom (N, P, or S) in the cation in the crystal compared to its length as a standalone cation.

**Figure 5:**
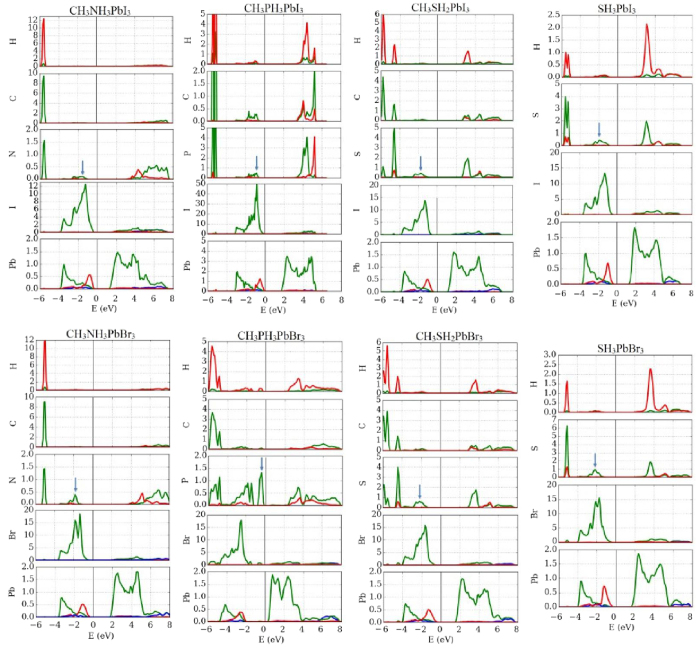
The projected density of states (PDOS) of all the studied materials (red for *s* orbitals, green for *p* orbitals, and blue for *d* orbitals). The arrows indicate the bridging states for which we calculate normalized PR values.

In the remaining of the paper, the focus will be toward the effects of electronic coupling on the stability. For that, we will compare CH_3_NH_3_^+^ cation with the new proposed ones; i.e. CH_3_PH_3_^+^, CH_3_SH_2_^+^, and SH_3_^+^. The most significant ramification of the enhanced electronic coupling affects the stability; the stable reaction and hull energies (Fig. 3) are considerably improved by a factor of –at least– 4 compared to CH_3_NH_3_PbI_3_ and CH_3_NH_3_PbBr_3_, except for CH_3_PH_3_PbBr_3_ which is completely unstable. As can be seen, both CH_3_NH_3_PbI_3_ and CH_3_NH_3_PbBr_3_ are marginally stable with reaction energies ranged between −25 and −30 meV/atom. In such case, the thermal energy at moderate temperature (50–100 °C) is enough to decompose the materials. Such decomposition does not happen spontaneously due to some kinetic barrier^10^. For all the other materials, the calculated reaction and hull energies stand between −230 and −100 meV. Worth-notingly, this behavior is correlated with the elongation of the bridging bond between the H atom and its donating atom in the cation (Fig. 4) compared to the standalone molecule^36^,^37^. The relevant bridging bond is between the electron donating element in cation (P, N, S) and its farthest H atom forming a bridge with the halide atom (I, Br) are reported. In the cases of CH_3_NH_3_PbI_3_ and CH_3_NH_3_PbBr_3_, the bonds are barely stretched by 0.97% and 0.87% respectively. This is mainly due to the high electronegativity of the N atom, which keeps the bonded H atoms strongly gripped. In term of normalized PR (Table 1), the contributions from both of them to the bridging states (indicated by arrows in Fig. 5) is small, implying that the associated H atom is highly localized within the cation and almost not interacting with the octahedron. On the other hand, the bridging P-H and S-H bonds are elongated much further and their corresponding normalized PRs are –at least– doubled. For the stable materials, the elongation (Fig. 4d) ranged between 2.1% and 4.4%, which is mainly due to the attraction of H atom to the halide and the bridge formation. So, the electronic densities of P, S, and H are delocalized due to the elongation and hence their normalized PRs are increased. This attraction becomes even more substantial in CH_3_PH_3_PbBr_3_ where the bond is 9.1% stretched. In this particular case, the bridging H atom is attracted significantly by the halide till it breaks the balance around it. We attribute this phenomena to the electronegativity difference between Br (2.96) and P (2.19)^38^ among others such as partial charges and polarizability (Fig. 1e).

The final relaxed geometry is sensitive to the starting orientation of the molecular cations. After full geometry optimization, the system often relaxes to the closest local minimum and finding the global minimum might be tedious. Therefore, there are several local minima connected with each other for the various probabilities of cation orientation. It was previously demonstrated that for CH_3_NH_3_PbI_3_, the various minima have energy difference of the order of ~10 meV or less^5^,^39^. To demonstrate this effect, we conducted an additional optimization from initially randomized cation positions for CH_3_SH_2_PbI_3_ (as shown in Figure-S6 in the Supplementary Materials); the total energy difference between the resulting fully relaxed structure and the structure used to evaluate the stability of CH_3_SH_2_PbI_3_ (in Fig. 1) which was relaxed from a more ordered cation orientation is less than 7 meV per atom. Hence, this energy difference is insignificant to alter the chemical stability of the compound in terms of the reaction and hull energies. Worth noting that the starting configuration has insignificant octahedral tilting but the two relaxed structures exhibit an octahedral titling of about 5° which indicate the strength of the hydrogen bonding. Lee *et al*.^40^ demonstrated that the octahedral tilting is governed by the strength of the hydrogen bonding in a great agreement with the results presented in this work.

So far, we mainly discussed the structural effects of the enhanced electronic coupling between the cation and the octahedron. Further understanding requires a close look at the electronic structure. Figure 5 show the projected density of states (PDOS). For CH_3_NH_3_PbI_3_ and CH_3_NH_3_PbBr_3_, the contributions of CH_3_NH_3_^+^ are deep in the valence and conduction bands. There are barely no signs for interactions between the cation and PbI_6_ and PbBr_6_ octahedra. This is severely altered by replacing by the suggested cations. Several molecular states caused by the enhanced electronic coupling appear at the top of the valance band. In the extreme case of very strong interaction, it results in emerging states occupying the top of the valance band and considerably shifting its edge as in the case of CH_3_PH_3_PbBr_3_ (Fig. 5).

**Figure 6:**
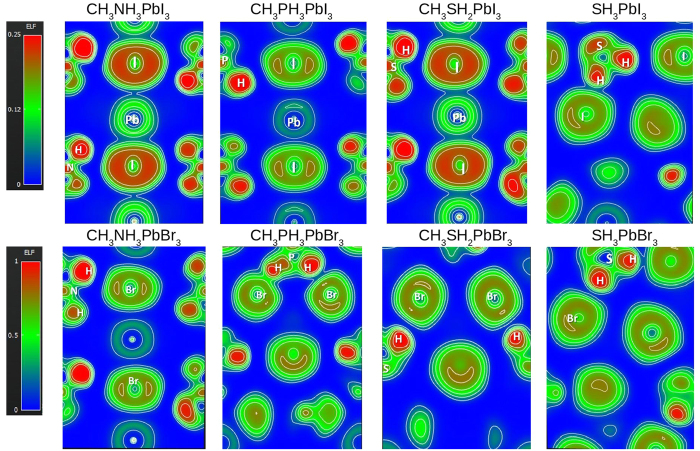
The contour maps of the electronic densities of the bridging states at planes with maximum interaction.

To visualize the electronic localization, the contour maps of the electronic densities of the bridging states at the planes with maximum interaction are plotted and shown in Fig. 6. In the case of CH_3_PH_3_PbBr_3_, it is clear that a very strong and connected bridge is formed as typical for hydrogen bonding. For SH_3_PbI_3_ and SH_3_PbBr_3_, the couplings between SH_3_^+^ cation and the halides are reasonably strong; but not to the level needed to form hydrogen-bond.

## Conclusion

In conclusion, we show how to chemically stabilize the hybrid perovskite solar cell absorbers by replacing methylammonium cation (CH_3_NH_3_^+^) by other cations that enhance the electronic coupling between the molecule and the octahedra while maintaining the band gap energy within the suitable range for solar cells. Practically, this is attainable by exploiting the electronegativity of the materials’ constituents and the resulting electrostatic interactions. Our calculations show that the stability is correlated to balancing the interaction and hence the electronic coupling between the halides in the octahedron and the cations. In this work, we considered CH_3_PH_3_^+^, CH_3_SH_2_^+^, and SH_3_^+^ cations where the reaction and hull energies are enhanced by a factor of –at least– 4; however, several other molecular cations can be used where the coupling and hence the stability can be enhanced.

## Methods

We employ density functional theory (DFT) calculations to evaluate the electronic structure and estimate the stability of the proposed materials. The DFT calculations are performed with the projector augmented wave (PAW) method as implemented in the Vienna Ab-initio Simulation Package (VASP)^41^. For the exchange correlation energy of interacting electrons, the generalized gradient approximation (GGA) with the parameterization of Perdew–Burke–Ernzerhof (PBE)^42^ is used. The energy cutoff for the planewave basis set was set to 520 eV and an 8 × 8 × 8 Monkhorst–Pack *k*-point mesh is employed where the convergence on the final forces is set at 0.01 eV/Å. VASP’s output also provides directly the participation ratio.

For the stability calculations, the phase diagrams (shown in the Supplementary materials) are generated for a given stoichiometry using PyMatGen with the Material project (MP) DataBase^33^, from which we deduce the decomposition route to the most stable binary and ternary compounds as explained in the Supplementary materials. Then, we calculate the reaction and hull energies for this decomposition route. The convex-hull^30^,^31^,^32^ construction method effectively evaluates the stability of a given compound against any linear combination of compounds that have the same averaged composition (the details are shown in the Supplementary materials). A material is considered stable only if the total energy difference between this material phases and the most stable alternative combination of reference systems is negative^30^. Also, to account for the calculation errors, the stability is considered marginal if the magnitude of the calculated energy is below some demarcation lines^29^,^30^,^31^,^32^,^43^. In this work, 100 meV/atom^43^ is assumed the demarcation line.

## Additional Information

**How to cite this article**: El-Mellouhi, F. *et al*. Enhancing Intrinsic Stability of Hybrid Perovskite Solar Cell by Strong, yet Balanced, Electronic Coupling. *Sci. Rep.* **6**, 30305; doi: 10.1038/srep30305 (2016).

## Supplementary Material

Supplementary Information

## Figures and Tables

**Table 1:** The Normalized participation ratios for the bridging states between the cations and the octahedra.

	N, P, or S	H	I or Br
CH_3_NH_3_PbI_3_	0.018	0.009	0.973
CH_3_NH_3_PbBr_3_	0.049	0.005	0.946
CH_3_PH_3_PbI_3_	0.068	0.034	0.898
CH_3_PH_3_PbBr_3_	0.694	0.056	0.250
CH_3_SH_2_PbI_3_	0.073	0.018	0.909
CH_3_SH_2_PbBr_3_	0.076	0.017	0.907
SH_3_PbI_3_	0.124	0.013	0.863
SH_3_PbBr_3_	0.202	0.011	0.787
