# Supplementary material for: Enhancing Intrinsic Stability of Hybrid Perovskite Solar Cell by Strong, yet Balanced, Electronic Coupling
Source: Sci Rep. 2016 Jul 26;6:30305. doi: 10.1038/srep30305 (PMC4960530; doi:10.1038/srep30305)
Supplement: Supplementary Information [file srep30305-s1.pdf]

Enhancing Intrinsic Stability of Hybrid Perovskite Solar Cell by Strong, yet Balanced, Electronic Coupling

Fedwa El-Mellouhi[1], El Tayeb Bentria[1], Sergey N Rashkeev[1], Sabre Kais[1,2,3], and Fahhad H Alharbi[1,2,*]

[1]Qatar Environment and Energy Research Institute (QEERI), Hamad Bin Khalifa University, Doha, Qatar

[2]College of Science and Engineering, Hamad Bin Khalifa University, Doha, Qatar

[3]Department of Chemistry, Physics, and Birck Nanotechnology Center, Purdue University, West Lafayette, Indiana 47907, USA

[*]falharbi@qf.org.qa

Supplementary Materials

COMPUTATIONAL DETAILS:

In this part, we present how to choose the most relevant reaction to phase stability for a given ternary, quaternary, or quinary phase. We use phase diagrams generated by PyMatGen using Materials Project data [1]. This method is based on the convex-hull construction reported in [2], which assumes that such construction effectively evaluates the stability of a given stoichiometry against any linear combination of compounds that have the same averaged composition. This procedure is one of the commonly used procedures to assess if a compound is stable versus the decomposition to other products. Ideally, a material is considered stable only if the total energy difference between this material phases and the most stable alternative combination of reference systems is negative [2].

To obtain the most stable combination, we used the following procedure. First, we plotted the phase diagram of the consisting elements "we add the element in the phase diagram if predicted", and then we detected which phases constitute the equilibrium triangle in which the material composition lies. The reaction from these phases in the equilibrium triangle to form the material phase is defined as the reaction critical to phase stability [3]. Each combination was fully relaxed and calculated using adequate parameters in conformity with the materials project data.

In the following subsections, we present the most relevant reactions to the phase stability for each the studied materials and show their location in the phase diagram.

$CsPbI_3$

The phase stability reaction of the $CsPbI_3$ is given by:

$CsPbI_3 = PbI_2 + CsI$

We can assess the decomposition reaction by the phase diagram of elements or simply the Hull construction.

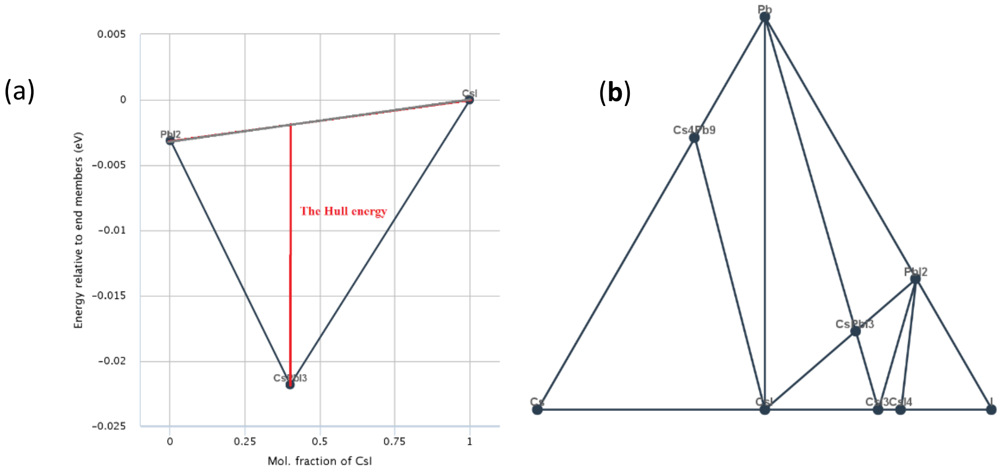

Figure S1: *The convex Hull of 3 element compound, (a) ternary phase diagram of Pb-I-Cs,(b) Convex hull construction for a CsI-PbI_2 system, where the y-axis is the formation energy per atom and the x-axis is the composition. Phase diagram and Convex hull construction are generated from [5].*

Table S1: *The reaction formation and Hull energies of $CsPbI_3$ in three phases. Energies are per atom.*

	Delta phase	Cubic	Tetragonal
Hull	-0.0908	0.0592	-0.0181
reaction	-0.0182	0.0118	-0.0036
Formation	-1.0456	-1.0156	-1.0311

SH_3PbI_3 and SH_3PbBr_3

The phase stability reactions of the **SH_3PbBr_3** and **SH_3PbI_3 are** given by:

SH_3PbBr_3 → PbS + 3 HBr

SH_3PbI_3 → PbS + 3 HI

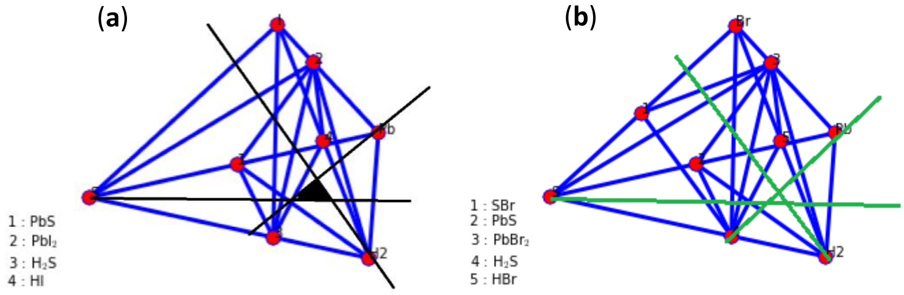

Figure S2: *The Hull triangle for 4 element compound, the black (green) lines intersection refer to the perovskite location in the phase diagram. (a) Br-Pb-H_2-S phase diagram, (b) I-Pb-H_2-S phase diagram.The lines in the quaternary phase diagrams define polyhedral with 4 vertices each, rather than triangles. At any point in the phase diagram other than the stable nodes, the equilibrium phases are given by the vertices of the polyhedron bounding that composition [4]. We can see that the polyhedral shape in (b) for example contain 1 = (PbS), 4 = (HI) and H_2 which correspond the phase stability reaction. Phase diagram can be generated from [5].*

$CH_3NH_3PbI_3$ and $CH_3NH_3PbBr_3$

The phase stability reactions of the **$CH_3NH_3PbI_3$** and **$CH_3NH_3PbBr_3$** are given by:

$$CH_3NH_3PbI_3 \rightarrow NH_4I + H_2C + PbI_2$$

$$CH_3NH_3PbBr_3 \rightarrow NH_4Br + H_2C + PbBr_2$$

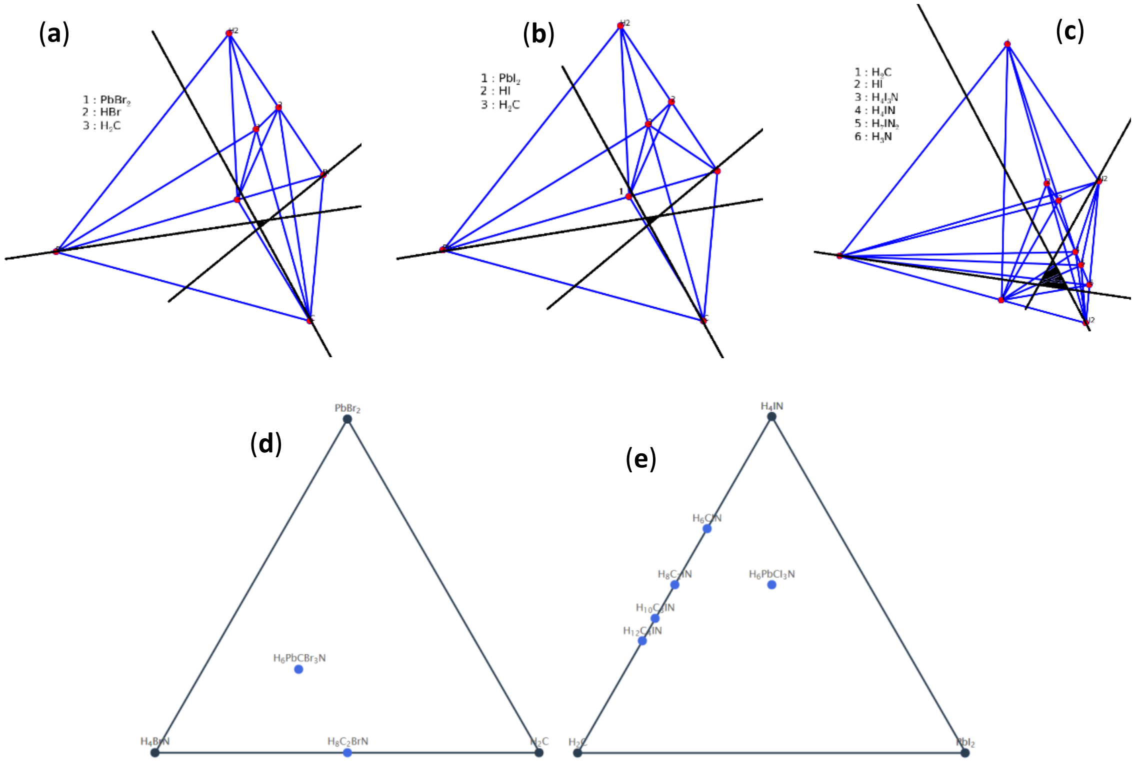

***Figure S3**: The Hull triangle for 5 element compound, the black lines intersection refer to the perovskite location in the phase diagram. We need to plot phase diagrams with different components for the same compound since MP can plot maximum of 4 atom type phase diagram, however grand potential phase diagram gives a clear insights of the phase stability compounds (a) C-H_2-I-N_2 phase diagram, (b) Pb-H_2-I-C, (C)Pb-H_2-Br-C. (d) H_4IN-PbI_2-H_2C compounds grand potential phase diagram, (e) $PbBr_2$-H_2C-H_4BrN compounds grand potential phase diagram. Phase diagram can be generated from [5].*

$CH_3SH_2PbI_3$ and $CH_3SH_2PbBr_3$

The phase stability reactions of the **$CH_3SH_2PbI_3$** and **$CH_3SH_2PbBr_3$** are given by:

$$CH_3SH_2PbI_3 \rightarrow PbS + H_2C + 3\ HI.$$

$$CH_3SH_2PbBr_3 \rightarrow PbS + H_2C + 3\ HBr.$$

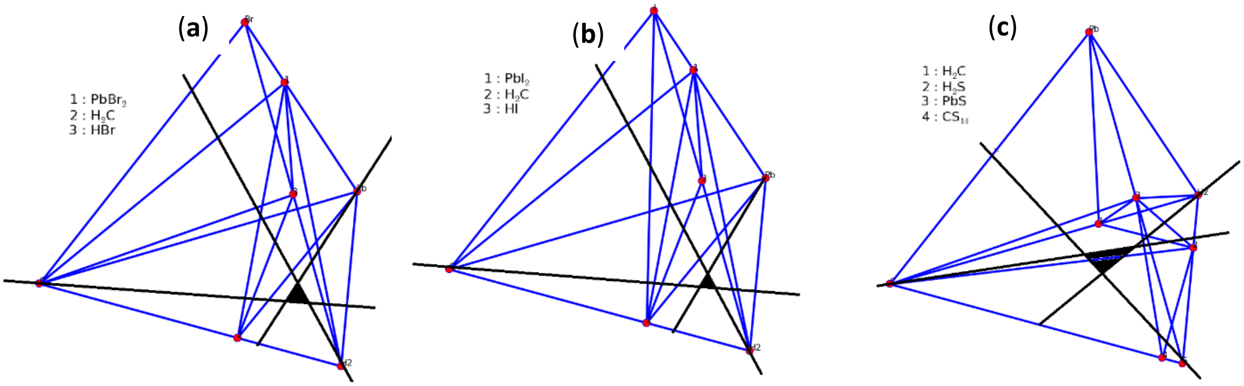

Figure S4: The Hull triangle for 5 element compound, the black lines intersection refer to the perovskite location in the phase diagram. (a) Pb-H_2-S-C, (b) I-Pb-H_2-I, (c) Br-Pb-H_2-C phase diagrams. Phase diagram can be generated from [5].

$CH_3PH_3PbI_3$ and $CH_3PH_3PbBr_3$

The phase stability reactions of the **$CH_3PH_3PbI_3$** and **$CH_3PH_3PbBr_3$** are given by:

$CH_3PH_3PbBr_3 \rightarrow PH_4Br + H_2C + PbBr_2$

$CH_3PH_3PbI_3 \rightarrow PH_4I + H_2C + PbI_2$

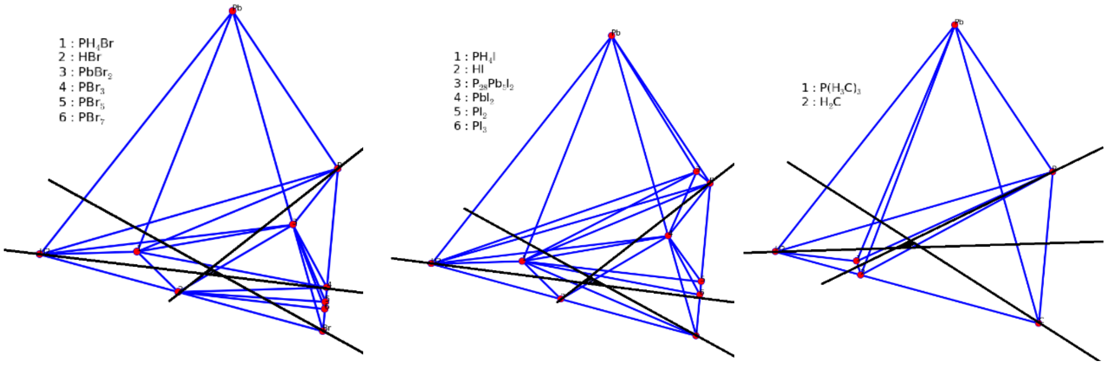

Figure S5: The Hull triangle for 5 element compound, the black lines intersection refer to the perovskite location in the phase diagram, (a) Pb-P-C-H_2, (b) Pb-P-I-H_2, (c) Pb-P-Br-H_2. Phase diagram can be generated from [5].

$CH(NH2)_2PbI_3$

The phase stability reaction of the **$CH(NH_2)_2PbI_3$** is given by:

$3\ CH(NH_2)_2PbI_3 \rightarrow 3\ C + NH_3 + 3\ PbI_2 + 3\ NH_4I + N_2$

where the phase stability reaction is extracted from Material project ID: mp-977014.

Table S2: The reaction formation and Hull energies of FAPI in three phases. Energies are per atom.

	alpha	delta
**reaction**	-0.03879	-0.04655
**Hull**	-0.05725	-0.06501
**formation**	-0.59922	-0.60698

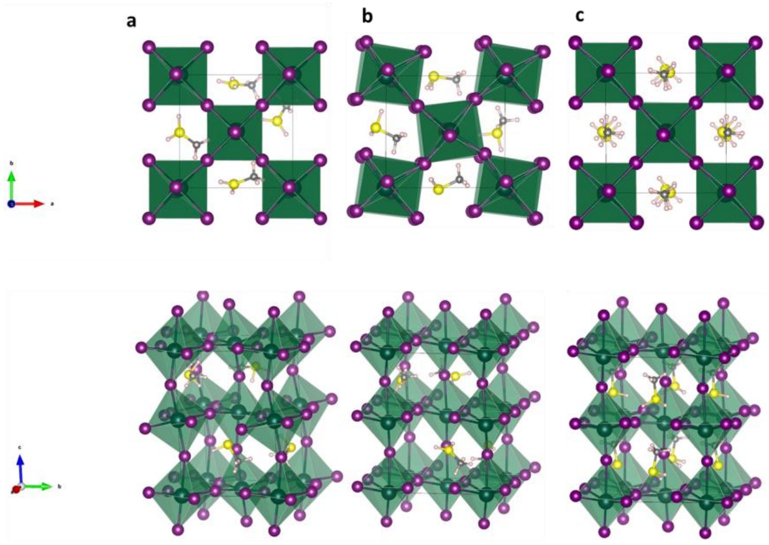

Figure S6: Initial randomized cation positions for $CH_3SH_2PbI_3$ (a) the resulting fully relaxed structure (b) and final structure starting from a more ordered cation orientation. Total energy differences between b and c are less than 7 meV per atom hence not altering the chemical stability of the compound.

REFERENCE

1. S. P. Ong, L. Wang, B. Kang, G. Ceder Li-Fe-P-O2 Phase Diagram from First Principles Calculations. Chemistry of Materials, 2008 20(5), 1798–1807. doi:10.1021/cm702327g.
2. Atahan-Evrenk, S., and Alan Aspuru-Guzik. "Prediction and calculation of crystal structures." Topics in Current Chemistry 345 (2014).
3. Hautier, Geoffroy, et al. "Accuracy of density functional theory in predicting formation energies of ternary oxides from binary oxides and its implication on phase stability." Physical Review B 85.15 (2012): 155208.
4. https://materialsproject.org/docs/phasediagram
5. https://materialsproject.org/#apps/phasediagram
